# Understanding Capacity to Treat First Episode Psychosis with a Hybrid Telemental Health Delivery Model: A Needs Assessment of Ohio Community Mental Health Centers

**DOI:** 10.1007/s10488-025-01437-y

**Published:** 2025-03-28

**Authors:** Brian P. O’Rourke, Jennifer L. Hefner, Nicholas J.K. Breitborde, Vicki L. Montesano, Kraig Knudsen, Tory H. Hogan

**Affiliations:** 1 The Division of Health Services Management and Policy, College of Public Health, The Ohio State University, 250 Cunz Hall, 1841 Neil Ave, 43210 Columbus, OH USA; 2 The Department of Psychiatry and Behavioral Health, College of Medicine, The Ohio State University, Columbus, OH USA; 3 Ohio Department of Mental Health and Addiction Services, Columbus, OH USA

**Keywords:** First episode psychosis, Coordinated specialty care, Community mental health centers, Telehealth, Health care delivery transformation

## Abstract

Coordinated specialty care (CSC) is considered the gold-standard treatment for individuals experiencing first episode psychosis (FEP). However, CSC teams are resource-intensive, motivating the development of a hybrid delivery approach where community mental health centers (CMHCs) collaborate with an academic medical center to deliver a mix of in-person and virtual services. To inform the development of this hybrid approach, a needs assessment was conducted, evaluating the existing capacity of Ohio CMHCs to treat FEP and identifying barriers to expanded use of telemental health. CMHC administrators throughout Ohio whose agencies primarily provide mental health services were surveyed using a novel instrument. A concurrent mixed methods approach combined multivariable analysis of cross-sectional survey data with thematic coding of responses to open-ended questions. The 56 responding CMHCs on average offered 10.96 of 17 services associated with CSC for FEP. Agency size was positively associated with number of service offerings, but rurality was not. Most agencies perceived gaps in their care for patients with FEP, particularly rural CMHCs. 75% believed that telemental health service expansion would benefit patients. Thematic analysis revealed three success factors for expanded telemental health usage: adapting care to virtual assessment, ensuring patient access, and adjusting workflows for virtual delivery. Responding CMHCs generally agreed that care for individuals with FEP could be improved and saw potential in expanded use of virtual services. Hybrid models may represent a valuable opportunity to overcome conventional barriers to CSC availability, but their development must account for current CMHC resource infrastructure and workflows.

**Supplementary Information:**

The online version contains supplementary material available at 10.1007/s10488-025-01437-y.

## Introduction

Psychotic disorders such as schizophrenia are long-term, serious mental illnesses that impact up to 3.5% of the US population (Kalin, 2023). The economic burden of schizophrenia alone in the U.S. is estimated to exceed $300 billion, which includes direct treatment expenditures and indirect costs such as loss of employment and caregiving needs (Kadakia et al., 2022). Considering these impacts, the early identification and treatment of individuals with psychotic disorders is a high priority (Cuthbert & Insel, 2010). However, the complex nature of the disease, stigma surrounding mental health care, and limited availability of evidence-based treatment in some areas all represent important barriers to care for individuals with psychotic disorders (Correll et al., 2010; Klosterkotter et al., 2011; Stokes et al., 2024; Thirthalli et al., 2017).

Recent research on psychiatric care models has identified coordinated specialty care (CSC) as an evidence-based treatment for individuals with recent onset of a psychotic disorder, a period that is called first episode psychosis (FEP) (Dixon et al., 2015; Read & Kohrt, 2022). CSC is a team-based approach to FEP with core services that include screening and intake, individual psychotherapy, medication management, case management, supported employment, supported education, and education/support for family and youth (Heinssen et al., 2014). The Recovery After an Initial Schizophrenia Episode initiative, funded by the National Institute of Mental Health, demonstrated that these teams are highly effective: patients who received CSC for FEP experienced reduced symptom burden and improvements in measures of social function that exceed those occurring under usual systems of care (Daley et al., 2020; Dixon et al., 2015). Subsequently, federal block grants became available for the development of CSC programs across the country, resulting in the widespread proliferation of this innovative care approach (George et al., 2022). In Ohio specifically, there were 18 active CSC teams across the state as of 2023, mainly housed in community mental health centers, covering 39 out of 88 counties (Breitborde et al., 2023). Given the rapid expansion of CSC, there has been a concerted effort to develop learning health networks, including the Early Psychosis Intervention Network, that promote the systematic collection of data to inform care improvements over time (Breitborde et al., 2023; Heinssen & Azrin, 2022). As such, there is a strong foundation for the implementation of CSC programs to ensure that people experiencing FEP have access to high-quality, evidence-based care.

Traditionally, CSC requires a multidisciplinary team of clinicians, frequently located in population-dense areas with substantial mental health human resources (Powell et al., 2021). As such, access to CSC for individuals with FEP is often geographically limited. The importance of geographic location is critical with this treatment modality because existing studies have documented that distance to psychiatric services is associated with delays in receiving CSC for FEP and reduced care engagement (Breitborde et al., 2008; Oluwoye et al., 2024). Additional factors, including stigma, patient disengagement, and securing consistent program funding, remain relevant as important potential barriers to the creation of CSC teams (Powell et al., 2021).

Community mental health centers (CMHCs), which serve thousands of patients across the country, are often the only provider available to treat complex mental health disorders like FEP in underserved communities. These organizations experience significant structural barriers that can make dissemination and implementation of evidence-based practices more challenging than other health care delivery settings (Editorial Board 2022; Hogan et al., 2022). The implementation of CSC programs normally requires CMHCs to employ a large, multidisciplinary team that includes a team leader, case manager, supported employment specialist, prescribers, nurses, support staff, and more, on top of incorporating these complex patients into their existing workflows (National Institute of Mental Health n.d.; Powell et al., 2021).

While some CMHCs can successfully adopt and sustain full CSC teams, one particular concern is workforce limitations, specifically in rural and health professional shortage areas (Powell et al., 2021). Even with sufficient funding, the ability to employ and retain the different staff members that make up a CSC team (including clinicians, social workers, and other support staff) remains an ongoing challenge. Additionally, allocating staff to CSC teams may not be possible at some CMHCs given the existing demand for behavioral health treatment in the community alongside relatively few FEP cases if the CMHC serves low-population, rural areas. Altogether, then, the ability to successfully curate a multidisciplinary team of mental health professionals within a single CMHC may be unrealistic in some areas. Considering this, mental health leaders have sought to find effective ways to support the implementation of evidence-based practices in rural CMHCs to close the evidence-to-practice gap in these underserved settings.

In response to the identified barriers to implementation, The Ohio State University Early Psychosis Intervention Center (EPICENTER) began developing a novel hybrid CSC for FEP delivery approach using funding from the Ohio Department of Mental Health and Addiction Services (OhioMHAS) (Breitborde et al., 2020, 2023). In this model, which is undergoing an implementation evaluation at two pilot sites using the RE-AIM framework (Gaglio et al., 2013), a team of psychiatrists and psychologists from EPICENTER will collaborate with a CMHC to provide a combination of telemental and in-person CSC services for FEP (Breitborde et al., 2023). This hybrid approach has the potential to enhance patient access to CSC for FEP around Ohio, even in areas with workforce challenges. However, amidst the specification and implementation of this model, it is critical to first understand the current capabilities of CMHCs to care for patients with FEP, as well as their perceptions about expanding the use of telehealth services. Additionally, the extent to which FEP treatment capacity differs across certain CMHC characteristics, such as rurality and overall size of the agency, has important consequences for the development of this model, and innovative mental health care delivery models more generally. For example, findings related to these organizational factors may demonstrate what types of CMHCs will benefit most from the implementation of the hybrid model.

To address these knowledge gaps, we conducted a needs assessment of Ohio CMHCs. The objectives of this study are to (1) examine the services that Ohio CMHCs have available for patients with FEP, as well as perceptions of current FEP treatment adequacy, (2) assess whether current FEP care capacity differs across agency size and rurality, and (3) identify attitudes about expanded telehealth service usage, including potential barriers to uptake. The results of this needs assessment will help ensure that the continued development and rollout of the hybrid CSC model is concordant with CMHC needs and can mitigate existing shortcomings in care for individuals with FEP. These findings are also informative for our broader understanding of mental health care delivery in context of building out new models for expanded treatment access.

## Methods

We surveyed administrators of CMHCs around Ohio to understand their current capacity to provide FEP treatment and their perceptions on expanded telemental health delivery. We utilized a concurrent mixed methods approach that combined descriptive analysis of cross-sectional survey data with thematic coding of responses to open-ended questions. The study was deemed exempt from review by The Ohio State University Institutional Review Board (IRB#2022E0410).

### Data collection

CMHCs, sometimes referred to as community behavioral health services providers, are defined as, “a community-based facility or group of facilities providing prevention, treatment, and rehabilitation mental health services” (American Psychological Association, 2018). OhioMHAS provided the research team with a list of all certified CMHCs, approximately 200, along with contact information for an agency administrator. Contacts for the CMHCs were emailed twice in October 2022 to complete the online survey. For this study, we only included CMHCs that primarily provide mental health services, as opposed to substance use services. This first screening question eliminated 48 of the original 112 respondents who completed the consent process. An additional eight respondents met the CMHC inclusion criteria but did not complete the survey, leaving a final sample of 56. Administrators who completed the full survey for their CMHC received a $20 gift card.

## Survey instrument

We designed a survey that examined the responding CMHCs’ current capacity to provide CSC for individuals with FEP as well as their perceptions about the feasibility of additional telemental health delivery. The instrument included five main sections: CMHC characteristics, FEP treatment offerings, perceptions of FEP treatment adequacy, current technological capacity, and open-ended perspectives on telemental health. Respondents were first asked to provide information on the characteristics of the CMHC, which included their location, number of patients served, total number of providers, and the types of communities mainly served (urban, suburban, rural, or a combination of these). Current FEP treatment services included the seven core offerings associated with CSC (Heinssen et al., 2014), as well as 11 other related services or activities associated with the EPICENTER CSC model (Breitborde et al., 2020), including availability of pharmacy services and treatments for comorbidities such as substance use. These services also align with treatment offerings that are part of the First-Episode Psychosis Services Fidelity Scale (Rosenblatt et al., 2024).

Perceptions of FEP treatment adequacy included level of agreement with the following statements on a 5-point Likert scale: (1) “the services currently provided at your agency meet the needs of individuals with FEP”; (2) “the services provided at your agency align with CSC for FEP best practice”; and (3) “there are gaps in the services available to meet the needs of individuals with FEP at your agency”. CMHCs were asked about whether they believed their patients would benefit from access to telephone-based and video-based services, and whether the following products were available for their patients to use: computers, webcams, secure videoconference software, microphones, and private office space.

In order to receive a more complete account of CMHC experiences with and attitudes about telehealth usage for the treatment of FEP, the survey instrument also included four open-ended questions. These questions focused on CMHC perceptions about concerns that could arise from additional telemental health utilization for patients with FEP, as well as organizational attributes that could promote or inhibit the overall use of CSC to treat FEP:

1. *What are your top three concerns about utilizing telephone-based services to treat individuals with FEP?* (46/56 provided a response)
2. *What are your top three concerns about utilizing video-based services to treat individuals with FEP?* (44/56 provided a response)
3. *What attributes does your agency have that may lead to your success for treating individuals within the context of CSC for FEP?* (43/56 provided a response)
4. *What are the barriers your agency faces related to treating individuals within the context of CSC for FEP?* (45/56 provided a response).

## Analysis

Agency characteristics were analyzed descriptively and compared across whether the CMHC had an active CSC for FEP team already in place, using Mann-Whitney U tests for the continuous variables, and chi-square tests for the categorical communities served variables. Measures related to FEP treatment offerings and perceptions of capacity to treat FEP patients were examined using regression analysis with two key independent variables, CMHC size and rurality. Logistic regression was used to examine whether the odds of offering any of the individual 17 services was associated with these independent variables. The association between these CMHC characteristics and the total count of FEP treatment offerings was analyzed as well with Poisson regression (Hayat & Higgins, 2014; Hutchinson & Holtman, 2005). For perceptions on system adequacy that were asked on a 5-level Likert scale, responses were collapsed into a dichotomous outcome (always/often vs. sometimes/rarely/never) and logistic regression was used.

Across these regression analyses, rurality was encoded using responses from the question about what types of communities the CMHC primarily served, which were any combination of urban, rural, and suburban. From these answers, a binary variable was constructed for rurality: “serves rural areas” and “does not serve rural areas”. These responses represent self-reported assessments of the areas and patients that the CMHC generally serves. Defining rurality is an ongoing challenge for health services research, with evidence that an individual’s subjective assessments of their community frequently differ from demographic classifications (Bennett et al., 2019). The issue is even more pronounced when attempting to classify the area that a provider serves, which will be even more varied. As such, we opted for a primary rurality classification based on administrator perceptions of the communities they served. To supplement this, an alternative analysis was performed with an existing classification of county-level rurality created by the Ohio Department of Health, where a rural county is defined as having a population of less than 50,000 with no urban cluster in or adjacent to it (Morrone et al., 2021). Using the list of counties that CMHCs described primarily serving, we determined whether each CMHC served at least one rural county. Results using this alternative measure are provided in the Appendix. CMHC size was measured on a scale of 100 patients served. While characteristics like number of staff and number of clinical sites were available, we used the number of patients as a proxy for overall CMHC size due to multicollinearity with these other variables (Morrow-Howell, 1994). These analyses excluded CMHCs that already had in-house CSC teams, given that these agencies should already be providing the full spectrum of CSC services.

Open-ended survey responses were analyzed using a two-stage inductive coding process. As previously described, the purpose of these open-ended questions was to get a more thorough account of how CMHC administrators perceived the potential benefits and pitfalls of additional telehealth usage, specifically in context of their ongoing care processes for patients with FEP. Responses were first ‘initial coded’: two authors (BPO, THH) created succinct codes for each response that stuck close to the data themselves (Skjott Linneberg & Korsgaard, 2019). Then, a second stage of ‘focused coding’ was used to draw out overarching themes related to the study objectives for these open-ended questions. When there were discrepancies with the core themes, the two authors used discussion to reach consensus on coding (Braun & Clarke, 2012). This process resulted in a series of findings that were integrated to create a set of considerations for achieving expanded use of telemental health delivery for patients with FEP, accounting for both telemental-health specific concerns as well as overarching barriers and facilitators to the incorporation of CSC for FEP at these agencies.

## Results

Administrators from 56 Ohio CMHCs completed the survey, representing an estimated 30% of CMHCs in Ohio. Responding agencies had an average of 3,556 patients, 110 staff members, and 3.8 clinical sites (Table 1). Half of all CMHCs reported serving nonrural areas only, and 21% served rural areas only. Agencies with an existing CSC for FEP team were significantly larger in terms of patient load, staff size, and number of clinical sites (*p* < 0.001).

**Table 1:** Characteristics of participating CMHCs

Characteristic	TOTAL	Does not have CSC for FEP team	Has CSC for FEP team	*p*
	*N* = 56	*N* = 48	*N* = 8	
*Patients served (mean*,* SD)*	3556.3 (7291.0)	1641.5 (2496.3)	14805.4 (14138.3)	***
*Number of staff (mean*,* SD)*	109.9 (182.4)	61.6 (90.7)	393.4 (308.4)	***
*Number of clinical sites (mean*,* SD)*	3.8 (4.4)	2.8 (2.5)	9.5 (2.8)	***
*Communities served (n*,* %)*				
Rural only	21.4%	22.9%	12.5%	
Rural and nonrural	28.6%	22.9%	62.5%	
Nonrural only	50.0%	54.2%	25.0%	

*p < 0.05; ** = *p* < 0.01; *** = *p* < 0.001

Table 2 shows the proportion of CMHCs without an active CSC for FEP team that offer each of the 17 FEP treatment services, which includes the seven core CSC team services as well as 11 additional FEP care modalities. Individual service availability within the sample ranged from 97.9% for *Screening and assessment/intake* to 25.5% for having an *Assertive community treatment team*. Controlling for rurality, larger agencies had significantly higher odds of offering two of the core CSC for FEP services: *Medication management* (*p = 0.02)* and *Supported employment* (*p = 0.04)*. Additionally, larger agencies had significantly higher odds of offering all four pharmacy-related treatment services. Controlling for agency size, CMHCs serving rural areas had somewhat lower odds of offering *Supported education* (*p = 0.07)* and having a *Pharmacy on site* (*p = 0.09)* than those that did not serve rural areas, at a marginally significant level. Finally, the bottom of Table 2 demonstrates that CMHC size is positively associated with the total number of FEP services they provide. CMHCs offered an average of 10.96/17 services. For each additional 100 patients served, the expected number of offerings increased by 0.58% when rurality was held constant (*p* < 0.001). Serving rural communities was not associated with the number of total offerings in these models. In other words, CMHCs that reported serving rural areas did not offer significantly fewer FEP treatment services than agencies serving urban and/or suburban areas. These findings are corroborated when using an alternative definition of rurality based on the counties served by the CMHC (see Appendix, Table 4). Additionally, this lack of association remains when using an alternative “serves *only* rural areas” versus “all other agencies” variable (results not shown).

**Table 2:** CSC for FEP service offerings amongst CMHCs without an active CSC team

FEP offering	Percent offering service	CMHC size (per 100 patients) adj. OR	*p*	Serves rural areas adj. OR	*p*
*Core CSC services*					
Screening and assessment/intake	97.9%	N/A		N/A	
Education and support for family members	93.5%	0.98		0.39	
Individual psychotherapy	93.5%	1.91		1.18	
Case management	91.1%	1.1		2.46	
Medication management	56.2%	1.12	*	0.91	
Supported education	48.9%	1.01		0.32	
Supported employment	29.8%	1.04	*	0.39	
*Additional related services-other treatment*					
Group psychotherapy	86.7%	1.22		1.50	
Crisis management	70.8%	1.01		0.34	
SUD treatment	70.2%	1.04		1.87	
Outpatient substance use treatment	69.6%	1.05		2.80	
Peer support	47.8%	0.99		0.36	
Assertive community treatment (ACT) team	25.5%	1.01		0.26	
*Additional related services-pharmacy*					
Long-acting injectable antipsychotic medication	45.8%	1.10	**	0.52	
Prescription assistance	50.0%	1.05	*	0.60	
Prescription of Clozapine	45.8%	1.06	*	0.68	
Pharmacy on site	28.9%	1.13	**	0.12	
*Total services offered*		*IRR*		*IRR*	
Mean services	10.96	1.006	***	0.93	

*p < 0.05; ** = *p* < 0.01; *** = *p* < 0.001

Table 3 displays measures related to perceptions on FEP treatment adequacy and benefits of telemental health delivery, as well as current telehealth-related technological resources available at the CMHC. Only about half of CMHCs without a current CSC for FEP team indicated that their services always/often met the needs of individuals with FEP (54.3%) and align with CSC for FEP best practice (50%). Similarly, more than 40% indicated there were always/often gaps in services available to meet the needs of individuals with FEP. Logistic regression analyses suggest that CMHCs who described serving rural areas/communities were significantly more likely to identify that there were always/often gaps in service availability (*p* = 0.048), and significantly less likely to believe that services always/often aligned with CSC for FEP best practice (*p* = 0.04). In secondary analyses with an alternative definition of rurality, the direction of these effects is the same, but they do not retain statistical significance (see Appendix Table 5). Approximately 75% of agencies saw benefits to expanded telephone-based and video-based service availability, which did not differ by CMHC size or rurality. Finally, technological capacity for telemental service delivery was mixed - most agencies had secure video conference software (85.4%) but only about half had webcams (46.8%), computers (52.1%), and microphones (56.2%) for patient use.

**Table 3:** Perceptions of FEP treatment capacity and technological capabilities amongst CMHCs without an active CSC team

	Percent	CMHC Size (per 100 patients)	*p*	Serves rural areas	*p*
*Percent Responding…*		adj. OR		adj. OR	
…that the services currently provided always/often met the needs of individuals with FEP	54.3%	1.03		0.37	
…that the services provided always/often aligned with CSC for FEP best practice	50.0%	1.02		0.25	*
…there are always/often gaps in the services available to meet the needs of individuals with FEP	42.6%	0.96		3.83	*
…that individuals with FEP would always/often benefit from access to telephone-based services	75.0%	1.00		0.43	
…that individuals with FEP would always/often benefit from access to video-based services	74.4%	1.00		0.37	
*Telemental Health Tech Resource Availability*					
Secure videoconference software	85.4%	1.00		0.39	
Private office for patients to use telemental health services	66.7%	1.03		1.38	
Microphone for patient use	56.2%	1.01		1.11	
Computer for patient use	52.1%	1.02		2.16	
Webcam for patient use	46.8%	1.02		1.62	

*p < 0.05; ** = *p* < 0.01; *** = *p* < 0.001

**Fig. 1:**
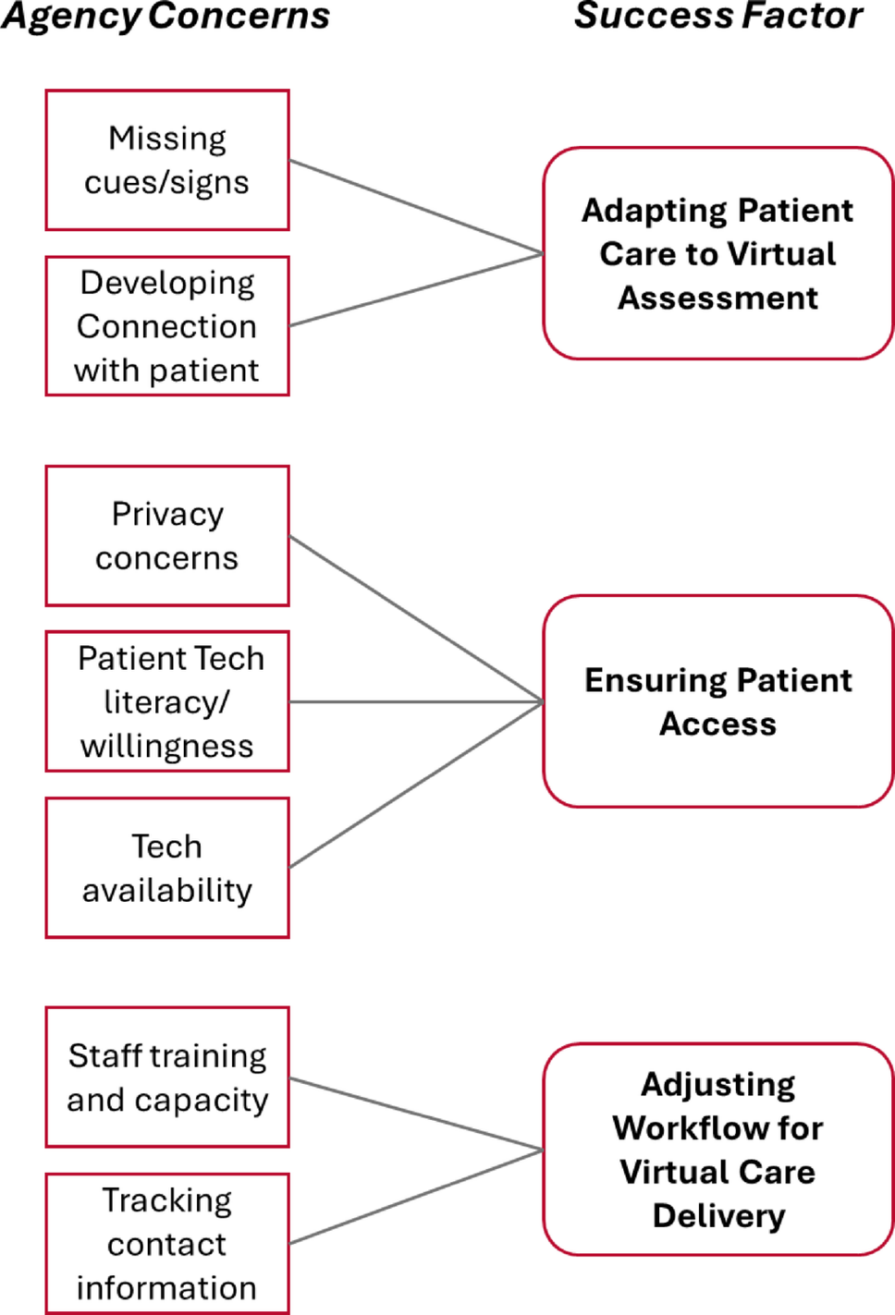
Considerations for expanded virtual delivery of CSC for FEP

In open-ended responses, agencies corroborated the potential of telemental health care delivery for their patients with FEP while also raising a variety of important barriers and considerations to keep in mind. Following thematic analysis of these answers, we identified three ‘success factors’ for productive telemental health expansion (Fig. 1).

### Adapting patient care to virtual assessment

CMHC administrators frequently noted that while virtual delivery of services would be highly beneficial for people with FEP, it came with some important considerations, namely the potential to miss cues/signs of patient issues and difficulties developing a patient-provider relationship. For instance, one administrator suggested that “*you really don’t know what is going on. You must rely on self-report and clients are not the best reporters*.” Another noted the “*lack of personal touch*” of telemental health. While virtual service offerings can address some gaps in care, other areas will still require in-person treatment to ensure a comprehensive assessment of patients and to foster a trusting relationship with members of the care team.

### Ensuring patient access

Agency administrators raised various concerns about access limitations of telemental health care delivery for patients with FEP. Alongside apprehension regarding the disclosure of private health information, CMHC respondents also pointed to potential hesitation amongst this patient population, with one CMHC administrator describing a situation of a “*person with delusions of television talking to them being triggered by a video-session with clinicians*.” Additionally, a variety of respondents questioned whether clients had sufficient technological capacity, including internet connectivity, necessary equipment, and tech literacy, especially in rural areas. These points heighten the importance of ensuring that novel hybrid care delivery models for FEP include an emphasis on making technological infrastructure available to patients, potentially within the physical locations of CMHCs.

### Adjusting workflow for virtual care delivery

The last important success factor for implementation of expanded telemental health delivery is the necessity of adjusting CMHC workflows to accommodate virtual care. Some administrators noted that additional staff training would be needed to ensure compliance with regulations surrounding telemental health and to ensure best practices for this care modality. Others raised substantial concern about keeping track of patient contact information that would be necessary to connect virtually, such as phone numbers. Implementing telemental service offerings must therefore involve a simultaneous adjustment to CMHC workflows.

## Discussion

The evidence-base supporting CSC for FEP highlights the importance of understanding the current landscape of FEP treatment and prospects for expanding the availability of CSC. Our needs assessment of Ohio CMHCs demonstrates that agencies range widely in their capacity to care for patients with FEP. CMHCs without a current CSC team offered on average 11 of the 17 services associated with FEP treatment; 4.96 out of 7 core services and 5.27 out of the 11 additional services. CMHC size was significantly associated with offering more CSC for FEP services overall, along with higher odds of offering individual services such as medication management, supported employment, and pharmacy-based treatments. While the magnitude of these effects was relatively small in our regression models, the overall impact can be substantial given the wide variation in CMHC sizes, which ranged in our sample from serving just 10 patients to over 38,000. Interpreting our Poisson model, a CMHC serving 100 patients would be expected to offer 9.2 of the 17 CSC-associated services, while a CMHC serving 5,000 patients would be expected to offer 12.2. This suggests that an ‘economy of scale’ effect may exist for FEP treatment capacity (Long et al., 1985), in which only agencies of a certain size have the ability to offer all components of CSC, regardless of location. It could be that smaller agencies have particular potential to benefit from a hybrid CSC model where some services, which currently may not be available at all for many smaller CMHCs, are covered by a virtual team.

When controlling for agency size, serving a rural community was not significantly associated with number of service offerings, nor was it associated with lower odds of offering any of the individual treatment services. This was also true when using alternative definitions of rurality based on the list of counties each agency described serving. The finding here is notable, given that many studies have documented the particular strains that rural health care providers face, including not only workforce shortages but also less local funding and fewer clients (Hastings & Cohn, 2013; Maganty et al., 2023; Morales et al., 2020). Previous geospatial work in Washington state has also demonstrated decreased CSC accessibility in rural areas, particularly for those that are underserved (Oluwoye et al., 2022).

Despite minimal differences in FEP treatment offerings by rurality, we also found that CMHCs indicating that they serve rural communities were more likely to see their treatment for patients with FEP as inadequate and misaligned with best practices. Given that CMHCs who described serving rural communities did not offer significantly fewer services for FEP than non-rural CMHCs, one explanation for their more negative perceptions related to care adequacy and alignment with CSC best practices is that some FEP services may not be offered consistently or at a high level of quality at these agencies. Recent research has demonstrated that fidelity to CSC care modalities is associated with improved symptomatic and functional outcomes (Rosenblatt et al., 2024), and more work is needed to assess the quality of these programmatic elements rather than just the presence or absence of them. It could be that the aforementioned rural health care barriers manifest not as reduced number of treatment offerings but as less consistent or lower quality treatment, which could explain why rural CMHCs had more negative attitudes about current care adequacy for their patients with FEP.

Speaking more to perceptions of care adequacy, our findings demonstrate that Ohio CMHCs have room for improvement overall, with nearly half of responding CMHCs identifying that there were always/often gaps in FEP treatment at their clinics. Importantly, these perceptions about care deficiencies exist alongside the widespread belief that telemental health delivery would be beneficial to these patients: over 75% of agencies believed that telemental health services could help bridge these gaps, though the technological infrastructure is lacking. Our qualitative findings additionally lend insight into success factors that would aid in the implementation of expanded telemental health usage for FEP, some of which are corroborated by previous studies of implementing FEP programs (Cohen et al., 2023). For instance, Cohen et al. find that expansion of CSC programs often requires significant attention to new staff roles and workflows. Telemental health undoubtedly offers significant potential to alleviate existing geographic mental health disparities (Myers, 2019), but its implementation requires a deliberate approach that is mindful of current CMHC capacity and administrator concerns related to patient access, staff training, and more.

Together, these findings suggest that there may be significant value to a hybrid CSC delivery model being piloted by EPICENTER with funding from OhioMHAS, where some components of CSC for FEP are provided by the CMHC itself and others are administered by a centralized clinician team at a major medical center– linked by virtual team meetings. This hybrid model offers the benefit of expanded access across geographic areas while also retaining the critical localized role of CMHCs in providing patients with a familiar place to receive ongoing care and potentially access technological products like webcams. The ability to transition some service offerings to the virtual team may enhance resource availability at CMHCs, allowing them to focus more thoroughly on the services they are responsible for and increase care quality in these areas. This may be particularly important in rural areas where workforce constraints continue to limit overall capacity (Merwin et al., 2003).

The potential of this hybrid team approach to enhance the delivery of evidence-based mental health care is responsive to significant federal investment, including commitments to FEP from the federal government via the 10% set-aside for CSC funding within state mental health block grants (Heinssen et al., 2014), which is particularly important given the costs associated with CSC (Nawaz et al., 2024). Given the concurrent existence of insufficient capacity and the potential of a hybrid telemental health model, policymakers should continue pursuing innovative forms of behavioral health care delivery that will not only improve care for individuals with FEP but also remain cognizant of the current resource availability of CMHCs. The successful implementation of these innovations requires close attention to current practices and anticipated barriers, as well as attention to agency characteristics that may impact both capacity and care adequacy. This may be especially true in rural areas, where there is a long history of barriers to implementing care innovations (Jensen et al., 2020; McDonel et al., 1997).

This work has a few important limitations. First, there is no official list of state CMHCs that align with our inclusion criteria (which excluded agencies primarily providing substance use treatment). To address the possibility that our sampling frame did not capture all eligible CMHCs, we reviewed our sampling approach with experts from OhioMHAS, who approved our final contact list. Additionally, the final sample of 56 agencies restricted the statistical analysis. A larger national sample of CMHCs may have yielded more generalizable findings, though the focus on Ohio was deliberate given the objective of understanding FEP treatment capacity amidst the rollout of a hybrid CSC delivery approach in the state. The 30% response rate for this needs assessment is comparable to other surveys of mental health service providers (Hawley et al., 2009), but we cannot rule out the impact of nonresponse bias.

Finally, information about the CMHCs is self-reported and represents a cross-sectional account of current care functions. While the assessment of CSC service availability at these agencies is valuable for our understanding of the current FEP treatment landscape, the reported presence of a CSC component does not provide information on the fidelity or quality of this service (Rosenblatt et al., 2024). Furthermore, all measures of rurality used in this study rely on self-reported data, either in terms of assessing the types of communities that a CMHC tends to serve (used for the main measure), or the list of counties that the CMHC primarily serves (used to construct a measure based on urban-rural county classifications in Ohio). While there were some differences in the analytical results when using the secondary rurality measure, albeit not directionally, the self-reported data about communities served may be a better reflection of whether a CMHC works with rural patients. This is especially true because most counties will have a mix of rural and non-rural areas, meaning an overarching definition is likely not completely accurate (Bennett et al., 2019). Still, an optimal definition of rurality may center on understanding the full geographic composition of the patient population, which was not available in this study.

Future work will begin to explore the implementation process for hybrid delivery of CSC for FEP, eventually focusing on patient satisfaction and treatment outcomes. Indicators of program uptake and changes in perceptions of service adequacy can be monitored longitudinally to get a detailed indication of improvement over time. While this study focuses on the perceptions of CMHC administrators, whose perspective is valuable for informing the development of a novel care delivery model, it will be important to also gather information on service users, families, and CMHC providers/staff during the program implementation process. There may be particular benefit to employing an inductive, interview-based qualitative approach when gathering these perspectives, allowing various stakeholders to speak to both the existing care functions for FEP and potential impacts of a new hybrid CSC model.

## Electronic Supplementary Material

Below is the link to the electronic supplementary material.

Supplementary Material 1

Supplementary Material 2
