# Supplementary material for: Understanding Capacity to Treat First Episode Psychosis with a Hybrid Telemental Health Delivery Model: A Needs Assessment of Ohio Community Mental Health Centers
Source: Adm Policy Ment Health. 2025 Mar 28;52(3):584–93. doi: 10.1007/s10488-025-01437-y (PMC12134010; doi:10.1007/s10488-025-01437-y)
Supplement: Supplementary file 1 — Supplementary Material 1 [file 10488_2025_1437_MOESM1_ESM.docx]

**APPENDIX TABLE 4: CSC for FEP Service Offerings Amongst CMHCs without an Active CSC Team, Using Alternative Classification of Serving Rural Areas**

| **FEP Offering** | *Percent Offering Service* | *CMHC Size (per 100 patients)* | *p* | *Serves Rural Areas* | *p* |
| --- | --- | --- | --- | --- | --- |
|  |  | adj. OR |  | adj. OR |  |
| *Core CSC Services* |  |  |  |  |  |
| Screening and assessment/intake | 97.9% | N/A |  | N/A |  |
| Education and support for family members | 93.5% | N/A |  | N/A |  |
| Individual psychotherapy | 93.5% | 1.96 |  | 0.84 |  |
| Case management | 91.1% | 1.11 |  | 0.82 |  |
| Medication management | 56.2% | 1.10 | * | 2.01 |  |
| Supported education | 48.9% | 1.01 |  | 0.44 |  |
| Supported employment | 29.8% | 1.03 | * | 0.63 |  |
|  |  |  |  |  |  |
| *Additional Related Services- Other Treatment* |  |  |  |  |  |
| Group psychotherapy | 86.7% | 1.27 |  | 0.55 |  |
| Crisis management | 70.8% | 1.01 |  | 0.59 |  |
| SUD treatment | 70.2% | 1.04 |  | 1.31 |  |
| Outpatient substance use treatment | 69.6% | 1.04 |  | 2.20 |  |
| Peer support | 47.8% | 1.00 |  | 0.58 |  |
| Assertive community treatment (ACT) team | 25.5% | 1.00 |  | 0.67 |  |
|  |  |  |  |  |  |
| *Additional Related Services- Pharmacy* |  |  |  |  |  |
| Long-acting injectable antipsychotic medication | 45.8% | 1.10 | * | 0.89 |  |
| Prescription assistance | 50.0% | 1.04 | * | 1.41 |  |
| Prescription of Clozapine | 45.8% | 1.05 | * | 1.74 |  |
| Pharmacy on site | 28.9% | 1.11 | ** | 0.39 |  |
|  |  |  |  |  |  |
| *TOTAL SERVICES OFFERED* |  | *IRR* |  | *IRR* |  |
| Mean services | 10.96 | 1.006 | *** | 1.03 |  |
|  |  |  |  |  |  |
|  | *p < 0.05; ** = p < 0.01; *** = p < 0.001 | | | |  |

*NOTE: For this alternative definition of rurality, a CMHC is designated as serving rural areas if at least one of the counties that it reports to serve is classified as rural by the Ohio Department of Health.*
