# Supplementary material for: Understanding Capacity to Treat First Episode Psychosis with a Hybrid Telemental Health Delivery Model: A Needs Assessment of Ohio Community Mental Health Centers
Source: Adm Policy Ment Health. 2025 Mar 28;52(3):584–93. doi: 10.1007/s10488-025-01437-y (PMC12134010; doi:10.1007/s10488-025-01437-y)
Supplement: Supplementary file 2 — Supplementary Material 2 [file 10488_2025_1437_MOESM2_ESM.docx]

**APPENDIX TABLE 5: Perceptions of FEP Treatment Capacity and Technological Capabilities Amongst CMHCs without an Active CSC Team Using Alternative Classification of Serving Rural Areas**

|  | *Percent* | *CMHC Size (per 100 patients)* | *p* | *Serves Rural Areas* | *p* |
| --- | --- | --- | --- | --- | --- |
| *Percent Responding…* |  | adj. OR |  | adj. OR |  |
| ...that the services currently provided always/often met the needs of individuals with FEP | 54.3% | 1.03 |  | 0.47 |  |
| …that the services provided always/often aligned with CSC for FEP best practice | 50.0% | 1.02 |  | 0.52 |  |
| …there are always/often gaps in the services available to meet the needs of individuals with FEP | 42.6% | 0.97 |  | 1.23 |  |
|  |  |  |  |  |  |
| ...that individuals with FEP would always/often benefit from access to telephone-based services | 75.0% | 1.00 |  | 0.60 |  |
| ...that individuals with FEP would always/often benefit from access to video-based services | 74.4% | 1.00 |  | 0.53 |  |
|  |  |  |  |  |  |
| *Telemental Health Tech Resource Availability* |  |  |  |  |  |
| Secure Videoconference Software | 85.4% | 1.00 |  | 1.39 |  |
| Private Office for Patients to Use Telemental Health Services | 66.7% | 1.03 |  | 0.67 |  |
| Microphone for Patient Use | 56.2% | 1.01 |  | 0.76 |  |
| Computer for Patient Use | 52.1% | 1.02 |  | 1.54 |  |
| Webcam for Patient Use | 46.8% | 1.02 |  | 1.85 |  |
|  | *p < 0.05; ** = p < 0.01; *** = p < 0.001 | | | | |

*NOTE: For this alternative definition of rurality, a CMHC is designated as serving rural areas if at least one of the counties that it reports to serve is classified as rural by the Ohio Department of Health.*
